# Data governance in predictive toxicology: A review

**DOI:** 10.1186/1758-2946-3-24

**Published:** 2011-07-13

**Authors:** Xin Fu, Anna Wojak, Daniel Neagu, Mick Ridley, Kim Travis

**Affiliations:** 1 School of Computing, Informatics and Media, Richmond Road, Bradford, BD7 1DP, UK; 2 Syngenta Ltd, Jealott's Hill International Research Centre, Bracknell, Berkshire, RG42 6EY, UK

## Abstract

**Background:**

Due to recent advances in data storage and sharing for further data processing in predictive toxicology, there is an increasing need for flexible data representations, secure and consistent data curation and automated data quality checking. Toxicity prediction involves multidisciplinary data. There are hundreds of collections of chemical, biological and toxicological data that are widely dispersed, mostly in the open literature, professional research bodies and commercial companies. In order to better manage and make full use of such large amount of toxicity data, there is a trend to develop functionalities aiming towards data governance in predictive toxicology to formalise a set of processes to guarantee high data quality and better data management. In this paper, data quality mainly refers in a data storage sense (e.g. accuracy, completeness and integrity) and not in a toxicological sense (e.g. the quality of experimental results).

**Results:**

This paper reviews seven widely used predictive toxicology data sources and applications, with a particular focus on their data governance aspects, including: data accuracy, data completeness, data integrity, metadata and its management, data availability and data authorisation. This review reveals the current problems (e.g. lack of systematic and standard measures of data quality) and desirable needs (e.g. better management and further use of captured metadata and the development of flexible multi-level user access authorisation schemas) of predictive toxicology data sources development. The analytical results will help to address a significant gap in toxicology data quality assessment and lead to the development of novel frameworks for predictive toxicology data and model governance.

**Conclusions:**

While the discussed public data sources are well developed, there nevertheless remain some gaps in the development of a data governance framework to support predictive toxicology. In this paper, data governance is identified as the new challenge in predictive toxicology, and a good use of it may provide a promising framework for developing high quality and easy accessible toxicity data repositories. This paper also identifies important research directions that require further investigation in this area.

## Introduction

With the enormous growth of organisational data and various possible ways to access such data, more and more organisations have become aware of the increasing significance of governing their data. Data governance involves a set of processes to improve data consistency and accuracy, reduce the cost of data management and increase security for the available data [1-3]. A good data governance framework ensures that organisational data is formally managed throughout the enterprise and provides efficient access to accurate data and business intelligent tools for further analysis.

It is important to note that data governance is different from data management. Data governance complements data management, but never replaces it. In general, management is about the decisions organisation make and it also involves implementing such decisions. Governance concerns what decisions need to be made by whom to ensure effective management, while aiming to provide a structure for achieving these tasks [4,5]. In other words, governance covers not only the decision domains, but also accountability for decision-making. Take organisational data quality for example, data governance provides a structure for identifying who in the organisation holds the decision making right to determine the standards for data quality, which aspects of data quality need to be included, and how to ensure such standards are attained. On the other hand, data management involves determining the actual metrics which will be employed to assess the pre-defined data quality standards. This paper is mainly concerned with the data governance aspect.

If an organisation settles on a good data governance framework, people within that organisation can more easily and effectively create a clear mission, achieve clarity, maintain scope and focus, increase confidence of using the organisational data, establish accountabilities, and define measurable successes [1]. However, establishing such data governance framework is not an easy task. The characteristics of the data, even the data itself, can be quite different from various organisations. This makes it very difficult to set a unique framework to assess and govern organisational data. In addition, data governance requires the bringing together of diverse expectations and expertises from different departments across the enterprise to achieve agreed, consistent, transparent and repeatable set of processes that enable better data-related decision making.

In many domains of life sciences such as pharmacology, cosmetics and food protection, toxicity assessment at the early stage in a chemical compound discovery process is receiving increasing attention. Predictive toxicology aims to address this problem. The large number of publicly available data sources, development of computational chemistry and biology, and rapidly increasing number of *in vitro *assays have contributed to the development of more accurate QSAR predictive models. The new REACH legislation [6] would require more animal testing to register a new chemical compound, if no alternative methods are used. This requirement pushes scientific institutions to move towards the better use of existing experimental data. Utilisation of toxicity information in conjunction with modelling techniques contribute to reduce the number of animal tests and decreases the cost of the new chemical compound discovery process.

Developing an interoperable and extensible data governance framework for predictive toxicology is crucial and highly required. First, toxicity data includes information about chemical compounds, *in vivo *and *in vitro *experiments. This large amount of information is distributed through publicly available, and often overlapping data sources. Different data formats, incomplete information about experiments and computational errors increase inconsistency of the collected information and make data governance challenging.

Second, in predictive toxicology, Quantitative Structure-Activity Relationships (QSAR) models relate the chemical compound structures to their measured effect or activity [7,8]. The quality of chemical and toxicity data has an impact on the process of model development for extracting new information, prediction or classification. Thus, it is crucial to ensure the accuracy and consistency of data. Currently, this process involves manual data curation and expert judgements of the data content. Automated data quality assessment is still a challenge for predictive toxicology [9,10].

Third, predictive toxicology is a multidisciplinary subject and the development of its data governance framework requires cross-functional groups/organisations to bring together the expertise needed to make predictive toxicology data-related decisions. This makes data governance in predictive toxicology more important and more difficult.

This paper attempts to offer a brief overview of data governance development in predictive toxicology. In particular, some of the most significant and recent public predictive toxicology data sources are reviewed, as well as a discussion on their data governance aspects. This helps to bridge the significant gap in toxicology data quality assessment and hopefully will attract more attention to develop and refine data governance frameworks for predictive toxicology. Such frameworks will provide standards in data representation and easy access to good quality publicly available information. It will also support data quality assessment, common reporting formats and collaborations in knowledge exchange amongst various organisations. The remainder of this paper is organised as follows. In the next section, the main concepts and components of a proposed data governance framework are introduced and discussed. It sets the boundary for the toxicity data sources discussion, with regard to data governance aspects. Based on these components, some of the most significant current toxicity data repositories are reviewed according to their data governance aspects in Section: *Review of Public Data Sources Supporting Predictive Toxicology*. The final section concludes the paper and identifies important further work.

## Data Governance: Main Decision Domains

Data governance receives increasing interest due to its importance and advantages in governing the use of data within and outside an organisation. This is evident in that data governance has recently been given prominence in many leading conferences, such as TDWI (The Data Warehousing Institute) World Conference, DAMA (Data Management Association) International Symposium, DG (Data Governance) Annual Conference and MDM (Master Data Management) Summit.

In addition to this, some different general data governance frameworks [1,5,11,12] have been proposed by different organisations and researchers with an attempt to provide guidance in designing and developing effective data governance approaches. Different organisations may have their own focus on specific aspects of data governance. The above frameworks all aim to provide support for the most common areas of their particular interests.

**Figure 1:**
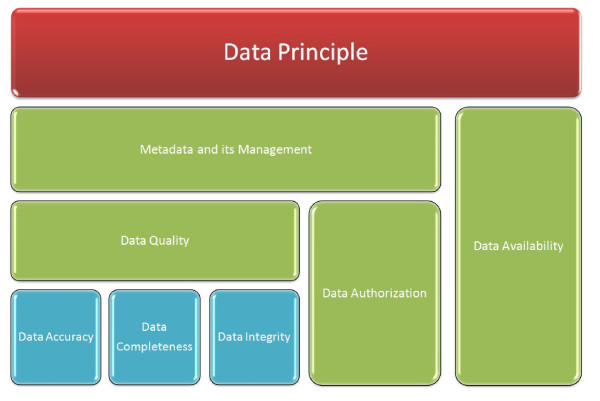
**Decision domains for data governance**.

It is obvious that due to the wide variety of backgrounds, motivations and expectations, the proposed general frameworks can be different. For example, the framework [5] which inherits an existing IT governance framework [13] places special interests in data principles and data life cycle, whereas the work of [11] focuses more on data warehouses and Business Intelligence (BI). Despite this, they still share some common decision domains, such as data quality, data availability and data privacy. In Figure 1, the main decision domains in predictive toxicology data governance that this paper focuses on are depicted. The scopes of the selected decision domains will be firstly introduced and these will serve as the boundary for the toxicity data sources and applications review provided in the following sections.

The data governance decision domains with particular interest for this paper include (see Figure 1):

• **data principle**: is the top level of data governance framework. An effective data principle provides a clear link between the data and the organisational business. Data principle establishes the goal and the main intended uses of data, thus it identifies the directions for all other decision domains. For example, the data principle identifies the data content and the standards for data quality, and these in turn are the basis for how data is interpreted by users in the format of metadata. Also, if the data principle will be publicly available, the data authorisation will be relatively simple.

• **data quality**: is one of the most important domains in data governance. Poor data quality inevitably has huge negative impacts on enterprise profits and operations. When it comes to predictive modelling, in order to develop accurate predictive models for toxicity values, high quality data is required. In a toxicological sense, data quality may refer to not only the recorded chemical information, but also the quality of experimental results. For example, whether the experiment was performed to Good Laboratory Practice standards (GLP) and whether the identify and purity of the tested chemical compounds were confirmed would affect the quality of experimental results. A more detailed discussion of data quality assessment in *in silico *toxicology can be found in [14].

Undoubtedly, poor quality chemical and toxicological data with errors and missing information contribute to poor predictive performance and low statistical fit [15,16]. This makes data quality checking a significant task and simultaneously a challenge for predictive toxicology. The quality of a study is judged based on its documentation, e.g. a study report or published paper. Therefore, in this paper, the quality of study data is considered in terms of data provenance under the *metadata and its management *domain. Rather than directly assessing the quality of experimental data, this paper more focuses on the discussion of whether the associated study metadata has been documented and provided together with data sources, which would help the users to make their own judgement about the quality of the underlying toxicology study.

Generally speaking, data quality refers to its fitness for serving its purpose in a given context [5]. In data storage sense, data quality often involves multiple dimensions, such as data accuracy, data integrity and completeness. In most cases, these dimensions need to be defined in the context of data usage. For predictive toxicology, the following three dimensions are of particular interest and they are defined as:

- **data accuracy**: is the fundamental dimension in data quality. Data may come from various internal and external sources, and therefore data accuracy refers to the correctness and consistency of data. For example, given the same chemical name, a poor quality chemical repository may return different chemical structures. This often confuses the user. A curation process is often involved to improve the data accuracy.

- **data completeness**: indicates that the required data are well recorded without missing values. A complete data source covers adequate data in both depth and breadth to meet the defined business information demand.

- **data integrity**: means the wholeness, entirety and soundness of organisational data [17]. It often involves a data integration process which combines data from different sources in an attempt to provide users with a unified view of these data. Data governance concerns some questions in this domain. For example, how to assure the integrity of data and how to determine if the integrity is compromised.

• **metadata and its management**: is defined as data which describes data. Understanding the data context as well as the content and encoding it into meta representation is a core aim of data governance [3]. A concise and consistent metadata representation makes the semantics of data become interpretable to users and ensures the data can be effectively used and tracked.

Organisational metadata can be classified into different categories, including the physical metadata, provenance metadata, domain-specific metadata and user metadata [5]. The physical storage metadata describes the physical storage of data. Provenance metadata refers to the data author and time stamp information, e.g. the source of the data, who is the owner of this data, when it was created and when has been last modified. If the recorded data is up-to-date for the task at hand and/or comes from a reliable source, it will contribute to better data quality. Domain-specific metadata summarises the semantics of data content. For example, in predictive toxicology, the study metadata indicates its key elements (e.g. study type, species and endpoints). User metadata captures the user information and historical usage record.

Different categories of metadata contain lots of valuable information and play key roles in data management, retrieval, discovery and analysis within organisations. The development of metadata repositories and their efficient stewardship is one of the essential activities of data governance and it will maximise the value of collected data, support decision making processes and business needs. In addition, due to the rapid changing business environment, the way an organisation conducts business and the consequent data also changes. As such, there is also a need to manage changes in metadata. Metadata management includes: data object standards and definitions, identifying relationships between data objects, providing accuracy, completeness and timeliness measurements, standards of documenting and reporting. In short, data governance uses metadata management to impose management discipline on the collection and control of data.

• **data availability**: concerns how users can access the data. It includes the data access and export formats (e.g. xml, pdf and spreadsheet), and how it can be accessed in what way and on what devices (e.g. PC and mobile phone). For example, some toxicity data sources can be downloaded in a dump sql file for local processing, whereas some data sources can only be queried about fixed fields via web/standalone system graphic user interface (GUI).

• **data authorisation**: controls user access to private and sensitive data based on user privileges. The data might have multi-level user access by the support of the pre-defined authorisation policies. The authorisation policy indicates what parts of the data can be accessed/manipulated by whom. For example, given a database, some data may be publicly available, while for some sensitive data, only authorised users can access it.

## Review of Public Data Sources Supporting Predictive Toxicology

**Figure 2:**
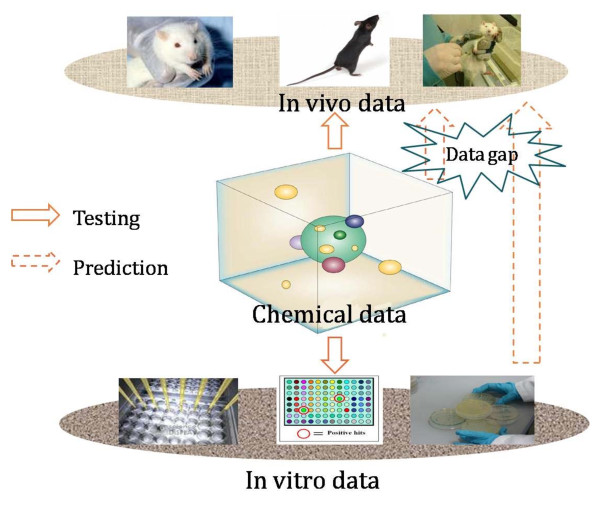
**Data domains for predictive toxicology**.

Collection of sufficient *in vivo - in vitro *assays is a starting point to build predictive models in predictive toxicity [15,18]. In the literature, there is a large amount of chemical, biological and toxicological data; however, only a small portion of them can be directly used for quantitative toxicity prediction. The current data sources mainly cover the following three major domains (see Figure 2):

• chemical data: this domain contains the chemicals and their associated information such as chemical descriptors, chemical and physical properties and molecular structures, which are used to build predictive models.

• *in vivo *toxicology data: refers to information collected from experiments or studies done in live organisms. Currently, *in vivo *assay data is distributed across various resources such as scientific articles, company internal reports, governmental organisation documents and many institutional services. Integrating this information in publicly available data sources by appropriate extraction, curation and pre-procession is challenging and extremely valuable.

• *in vitro *data: *in vitro *cell and molecular biology technology has attracted more attention of toxicology researchers, due to its relatively low cost. In comparison to *in vivo *technology, *in vitro *research is more suitable for the deduction of biological mechanisms of action [15]. Together with chemical information *in vitro *data are used to predict *in vivo *toxicity and to prioritise animal testing.

The huge amount of varied data impacts on the modelling process. However, there exists a common problem in data quality assessment. Due to the lack of standard measures (such as accuracy, consistency, comprehensiveness, coverage, accessibility, reliability) for quality evaluation, the quality of data sources is either not assessed or just assessed manually. The current assessment is normally quite subjective and mainly depends on assessors' own experiences and preferences. This makes the data governance of current toxicological data sources very important.

Recently, many toxicogenomics and chemical data sources have been reviewed according to needs and challenges in predictive toxicology [18-24]. In this section, the discussion of several major data sources that are widely applied to predictive toxicology is presented, with particular focus on their data governance aspects.

More specifically, in the context of data governance, the framework which has been proposed in the previous section sets the boundary for discussion. The main framework components, including data accuracy, data completeness, data integrity, metadata and its management, data availability and data authorisation are the main interest of this paper. Their status in different data sources will be discussed respectively. It is also worth noting that the list of data sources discussed here is not exhaustive, but the selected data sources illustrate well the range of the publicly available data sources. Table 1 details the analysis of various data sources according to the previously mentioned components of data governance framework.

**Table 1:** Discussion of public data sources in terms of data governance

Database	Data Accuracy	Data Completeness	Data Integrity	Metadata	Data Availability	Data Authorisation
ChemSpider	manual and automated data curation, crowd-sourcing community supported	claimed to be the richest source of structure-based chemistry	an aggregator of nearly 400 different data sources	includes data sources metadata, provenance metadata (e.g. owner and time-stamp of data creation, curation and update) and user meta-data	can be accessed by web GUI and web services via PC and mobile devices; query results can be downloaded as a set	publicly available, no multi-level user access supported

CEBS	manual data curation, collaboration between data de-positors and internal curation staffs	contains 132 chemicals and their response in 34 detailed studies	permits users to integrate various data types and studies, a database schema is well designed by the support of controlled vocabularies	includes domain-specific metadata (e.g. owner, study details) and provenance metadata (e.g. time-stamp of the study start, curation updates)	allows users to retrieve and combine customised information and export to various formats for downloading. Able to support up to 100 concurrent users	publicly available, but also provides private data access mode to protect sensitive user data

CTD	manual data curation, sup-ported by the scientific com-munity	includes 1.4 million chemical-gene-disease data connections and has been widely recognised	employs community-accepted vocabularies and ontologies to capture data and is integrated with external resources	includes domain-specific, provenance metadata and supporting literature sources are recorded	access to entire database (downloadable as a dump file) and individual data sources; query results can be customised and exported to different formats	publicly available, no multi-level user access provided

DSSTox	manual and automated data curation, quality assurance log files recorded; but no curation for external data	contains over 8000 chemicals and have been incorporated into several external sources	integrates molecular structures and toxicity data into standardised DSSTox SDF	includes domain-specific and provenance metadata	data sources and associated documents can be downloaded individually and included data is searchable via many options	publicly available, no user registration is required

ToxCast	manual data curation, includes internal and external review	covers various chemical classes and diverse mechanism of action, 320 chemicals have been collected in Phase I and 1000 more is currently being screened	well integrated into many other EPA databases	only domain-specific metadata is included, no recorded track of provenance metadata (e.g. curator and time-stamp)	data sources are available for download individually in the ToxCast website; included data can be browsed and queried via ToxCast DB web GUI	publicly available, no user registration is required

ACToR	does not provide any data curation itself, data quality totally depends on the original data sources	contains over 500,000 chemicals and associated toxicity data from nearly 500 sources	itself is a chemical toxicity data aggregator and employs a clear and flexible database schema for data integration	metadata of data source and domain-specific information is well recorded, no recorded track of provenance meta-data	open-source implementation and the entire database can be downloaded	publicly available, no user registration is required

OpenTox	allows for automated, man-ual and global data quality validations	contains different categories of public data sources which supporting predictive toxicology	employs ontology to support efficient integration of data coming from different sources into a unifying structure	metadata of data source and domain-specific information is well recorded, no recorded track of provenance meta-data	provides APIs and REST-ful web services for included data, algorithms, models, ontologies and reports	publicly available, but employs OpenSSO for initial implementation of multi-level user access

### ChemSpider

The development of the Internet fosters on-line publications of chemical information by various organisations. The broad distribution of chemical and physical properties and molecular structures results in duplicated storage cost, inefficient information retrieval and inconsistency. Thus, there was a need to provide a framework for data integration and access to high quality chemical information.

The free online repository ChemSpider [25] was developed to address this problem. It was launched in 2007 to provide searchable chemical structures and property information in the public domain. The key feature of ChemSpider is the compound structure centric database. All possible available information about a given chemical compound is linked by its molecular structure, though ChemSpider is not an exhaustive database. The aim of ChemSpider is to provide services in chemical information searching, and also to build a crowd-sourcing community. In predictive toxicology, this community is a group of specialists from different organisations that contribute their knowledge to improve the quality of collected information in a database. At present, ChemSpider does not directly support toxicity predictions, however, the content of ChemSpider provides high quality chemical information and links to original resources that are very useful for building QSAR models.

Reflecting back to the data governance framework (see Figure 1), the main domains are discussed as follows:

• **data accuracy: **ChemSpider has realised that ensuring the accuracy of included data is an essential requirement for public data sources, and it is trying to distinguish itself from other public chemical repositories (e.g. PubChem [26] from the National Institutes of Health (NIH)). The NIH currently lacks a data curation mechanism to ensure the quality of included data. It relies totally on the data depositors to curate their own data. As a result, errors from various depositors and multiple representations and formats are included in PubChem. Rather than depending on any individual depositor, ChemSpider has spent much effort in improving data quality by employing the crowd-sourcing activities of the community. The following systematically organised data curation is performed by ChemSpider and this can be achieved both automatically and manually:

- the general curation activities include: removing incorrect names, correcting spellings, adding multilingual names and alternative names.

- to avoid anonymous act of vandalism, only registered users are allowed to edit the records.

- when uploading a chemical structure to ChemSpider, automated chemistry checking is performed.

- domain experts are invited to continuously validate and update included data. The data curation labels include "validated by experts", "validated by users", "non-validated", "redirected by users" and "redirect approved by experts" [27].

Currently, more than 130 people are involved in data validation and annotation. Over a million chemical structure and identifier relationships have been validated either automatically or manually [22]. This data curation effort will continue with an intention to offer the highest quality online chemical database.

• **data completeness: **ChemSpider currently contains over 25 million compounds (ChemSpider Count 03/2011) and the number is growing daily. It is claimed to be the richest source of structure-based chemistry. The variety of information about a compound provided at ChemSpider is hard to match on any other free website.

• **data integrity**: ChemSpider aims to act as an aggregator of chemical information. Data from nearly 400 different data sources (including Wikipedia, PubChem [26], ChEBI [28] and etc) are integrated and linked by means of chemical structure. Where possible, each chemical record retains the links to the original source of the material and also links out to other information of particular interests, including where to purchase a chemical, chemical toxicity, metabolism data, etc. Instead of employing classical search engines (e.g. Google) to search individual pieces of information, aggregating such chemical information into a central database saves lots of search effort and time for users.

• **metadata and its management: **ChemSpider uses metadata representation to extract information about a chemical compound, its associated data sources together with its external IDs (if available) and relevant scientific articles. Additionally, the creation date and owners of the included data sources are provided in ChemSpider. The service keeps track of curation updates and makes this information available to the master curators. Also, once registered users login to the system to perform the search, their search history will be recorded and stored. This information can be further used to capture user preferences and then provide more customised services.

• **data availability: **ChemSpider also realised the importance of data availability and accessibility. The website provides various simple and advanced search options via a user friendly web GUI. In addition to this, the system provides web services (including APIs) to allow users to query the content and request physiochemical properties prediction, and the retrieved results can be downloaded as a set. The included structure images and spectra can be easily embedded into external web pages by the use of provided tools. In addition, the newly developed Mobile ChemSpider allows users to access ChemSpider data through mobile devices, such as mobile phone browsers and iPads. Note that users are limited to assemble 5000 ChemSpider records or less to build an in-house data source.

• **data authorisation: **ChemSpider is a publicly available database, the RSC logs the IP address of user's PC to be able to receive and send information on the Internet. However, no multi-level user access is provided, all registered users share the same authorisation schema to access the data.

### CEBS

Chemical Effects in Biological Systems (CEBS) is the first public repository which captures toxicogenomics data developed by the National Center for Toxicogenomics (NCT) within the National Institute of Environmental Health Science (NIEHS) [23,29]. A distinguishing feature of CEBS is that it contains very detailed animal-level study information including treatment protocols, study design, study time-line, metadata for microarray and proteomics data, histopathology and even raw genomic microarray results [23].

The objective of CEBS is to provide users easy access to the integrated wide diversity of data types and detailed study information. The embedded rich information makes it possible to develop answers to comprehensive queries posted in the database, and then conduct gene signature and pathway analysis based on the retrieved answers. Users are allowed to query the data using study conditions, subject responses and microarray module.

The main domains in the previously presented data governance framework (as shown in Figure 1) are described as:

• **data accuracy: **the accuracy of CEBS data is handled by collaboration between the data depositors and internal curation staff. Prior to being exported to CEBS database, all study information needs to go through Biomedical Investigation Database (BID) which is a component of CEBS responsible for loading and curating data.

• **data completeness: **as of 2010, CEBS contains 132 chemicals (structure searchable via U.S EPA(Environmental Protection Agency) DSSTox) and their responses which were derived from 34 studies in mice, rats, and Caenorhabditis elegans [18]. Most of the included studies have associated microarray data. CEBS welcomes high-quality study data relating to environmental health, pharmacology and toxicology. When submitting such data, a study design and phenotypic anchoring data is required.

• **data integrity: **one of the main objectives of CEBS is to permit users to integrate various data types and studies. By the support of a well designed relational database schema, the CEBS users can effectively retrieve the associated biological and toxicological data based on the selected subject response or study conditions, etc. CEBS has employed controlled vocabularies (i.e. CEBS data dictionary) rather than free text to capture study related data, allowing data to be integrated within a given microarray platform for effective filtering, query and analysis. On the other hand, CEBS has to manage data from a variety of resources and each resource may use different study designs, treatment regimes and measures. Currently, there is lack of a widely accepted public standard for exchange and capture of such study design and assay data. A number of effort is under way to address this need, and CEBS will fully support the development of a standard which will provide better data integration.

• **metadata and its management: **every study included in CEBS has its associated details document containing the following information: the institution, principal investigator, start date of the study, design details and supporting publications (together with their PubMed IDs). Such metadata can be easily retrieved in various graphical representations by clicking the provided links on the web page. This detailed domain-specific metadata helps to provide customised search options and then maximise the included data values.

• **data availability: **CEBS provides easy access for users to retrieve, combine and download customised information. By using the SysTox browser, the CEBS users can combine components of different workflows provided by CEBS to customise their queries and export them to various formats for downloading. Alternatively, users can use the provided FTP service to individually download data sources. In addition, users are allowed to create and manage their own workspaces. The infrastructure has been simulated to be able to support up to 100 concurrent users in various use cases and workflows.

• **data authorisation: **although CEBS is claimed as a public repository integrating study design and assay data, it only allows public access to the fully published data sources. For those not fully published data sources, they are not available for downloading without prior agreements of data owners. It is common to obtain access to unpublished data sources on a collaborative basis.

One unique feature of CEBS is its allowance for users to upload their own data into CEBS in a private mode and only make it visible to their nominated collaborators behind the CEBS firewall [30]. This private data authorisation schema securely protects sensitive user data and also allows users to integrate their own data with other public data sources in CEBS for analysis.

### CDT

Environmental chemicals may play a crucial role in the etiology of human diseases. Despite this observation, the mechanism of action and the potential influences of most chemicals on many diseases are not known [19,20,31,32]. To gain a better understanding about the impact environmental chemicals have on human health, the Comparative Toxicogenomics Database (CTD) [33,34] has been developed by Mount Desert Island Biological Laboratory. It serves as a unique centralised and freely available resource to explore the interactions amongst chemicals, genes or proteins and diseases in diverse species.

Chemicals might interact with various genes and proteins in multiple ways and then affect the mechanisms underlying the etiology of diseases. One of the major goals of CTD is to support the generation of novel hypotheses about chemical actions and environmental diseases. It is worth noting that CTD acts not only as a data repository but also as a discovery tool to generate novel inferences. The inferred interactions can also be evaluated based on local statistics, and the derived ranking scores will help users to prioritise further testing. In addition, a set of tools to visualise, manipulate and analyse different types of data such as comparisons of gene sequences from different species or comparisons of associated data sources for up to three chemicals, diseases or genes are provided by CTD.

In terms of data governance framework components, the following aspects are considered:

• **data accuracy: **the included chemical-gene interactions, chemical-disease and gene-disease relationships in CTD are completely manually curated, thus the data accuracy relies solely on professional experts. Curators should be trained in the data curation according to CTD requirements. Prior to monthly public releases, the curated data submitted by an individual curator still needs to go through a further review conducted by the scientific community, and this helps to ensure the high accuracy of curated data. It is obvious that such manual curation is time consuming and this task becomes more challenging with the increasing scope and volume of available data. With an attempt to speed up and improve the efficiency of manual data curation, a prototype text-mining application has recently been developed to prioritise the available literature [32].

• **data completeness: **to date, CTD database includes 1.4 million chemical-gene-disease data connections and new data is available monthly. It currently consists of over 240,300 molecular interactions between 5900 unique chemicals and 17,300 gene products, 11,500 direct gene-disease relationships and 8500 direct chemical-disease relationships extracted from over 21,600 publications [19]. The applicability and utility of CTD has been widely recognised and it is evident that CTD is being indexed by many public repositories.

• **data integrity: **all curated data, especially the inferred interactions, are captured in a structured manner to minimise the inconsistency amongst different curators. This is achieved by employing community-accepted controlled vocabularies and ontologies. This not only helps users to retrieve data efficiently, but also provides a useful way of integrating and communicating with external data sources by using consistent terms. CTD data is integrated with a number of external chemical, gene, disease and pathway resources, including ChemIDplus, DrugBank, Gene Ontology Consortium, NCBI Gene, NCBI PubMed, etc [20].

• **metadata and its management: **the original references of the curated interactions are included in CTD. For quality monitoring and follow-up purposes, the data of the curation such as the curator ID, date of curation and related articles is also recorded. Note that, the metadata of CTD records not just providing the domain-specific information, but also includes some evaluation metrix, such as similarity score and inference score.

• **data availability: **CTD provides a wide variety of ways to access the included data, users can easily download individual data sources. Alternatively, the whole database as a dump file can be downloaded for local analysis. In addition, CTD allows users to perform both detailed query and batch query to find various types of data instead of just those relating to a specific chemical, gene or disease term. The retrieved results can be customised and exported to different formats (such as csv, xml and tsv).

• **data authorisation: **CTD is a community-supported public resource tool that advances understanding of the effects of chemicals on human health. However, no multi-level user access is available. All registered users share the same authorisation schema to access the data, so that there is no authorisation protection of users' private data.

### DSSTox

The Distributed Structure-Searchable Toxicity (DSSTox) public database network provides a public forum for high-quality, standardised chemical structure files associated with toxicity data [35]. It aims to help in building publicly and easily accessible data collection to improve predictive toxicology capabilities. A major goal of the DSSTox project is to encourage the use of the DSSTox data format (including DSSTox Standard chemical structure fields and standardised SDF (structure data format)) for publishing chemicals and their associated toxicity data files.

Recalling the data governance framework in Figure 1, the related domains are discussed as:

• **data accuracy: **one of the unique features of DSSTox is the quality control of the included data. All documentation and data files considered for DSSTox publication are subject to DSSTox project review, EPA internal review, and in some cases outside peer review [35]. In addition, most data sources are curated by experts and uniformly applied into DSSTox [18]. DSSTox deals with the quality control process only according to the information stored in its own data sources. An extensive and clear information quality review procedure is applied to the annotation of included data. Every data source includes a quality assurance log file. This file summarises all undertaken procedures to ensure accuracy and consistency of chemical structure within a data source [35]. Chemical structures are retrieved from outside sources and cross-validated for internal consistency. All errors and missing values are reported in a log file. Moreover, DSSTox provides procedures for users to report errors in DSSTox data sources. Such community efforts support integration and migration of chemical toxicity information into DSSTox. Finally, data is validated by experts (DSSTox users). It is worth noting that DSSTox does not provide a quality review for information collected from outside resources.

• **data completeness: **the current DSSTox database contains over 8000 chemicals and has been incorporated into several external resources, including: ChemSpider, PubChem inventory [26], GEO [36], ACToR database, ArrayExpress Repository, and EMBL-EBI. DSSTox standardised format has gradually gained popularity in recent years. It is anticipated that more public chemical information and related toxicity data will be migrated into the DSSTox for publishing in the near future.

• **data integrity: **publicly available toxicity data sources exist in different locations and widely disparate file formats. They are quite different in terms of toxicity endpoints, test methods, treatment conditions and degrees of result details. Additionally, they are often not downloadable in their entirety and most do not include related chemical structures embedded with rich content. Being aware of this, DSSTox extends the existing SDF and annotates the list with DSSTox Standard Chemical Fields to integrate the molecular structures and toxicity data into standardised DSSTox SDF. This is fully incorporated into U.S EPA ACToR and PubChem.

• **metadata and its management: **metadata of published data sources are well recorded. Every data source is associated with documentation including following information: how and when chemical compounds were collected into a data source, links to the outside repositories, time-stamp of when a data source was created and updated, information about last update; a list of authors and reviewers of DSSTox data source and supporting literature.

• **data availability: **DSSTox data sources and their documentations can be downloaded from the DSSTox website. User contributions are used to build larger DSSTox user communities. Each of the published data files can be freely downloaded in common formats such as pdf, sdf and spreadsheet, and are of potential use for (Q)SAR modelling. Large files are offered in compressed file format. In addition, a DSSTox Structure-Browser has been developed by EPA to provide a simple and handy structure-searching capability. The full collection of DSSTox published data files is searchable using many options, including chemical text, data file, SMILES, structure, generic test substance level and outputs.

• **data authorisation: **DSSTox allows full and publicly open access to included toxicity data files and no user registration is required. Although the DSSTox website collects and stores user access log file, including access date and time, IP address, the objects/web page requested, access status and etc, no multi-level user access is enabled, all users share the same authorisation schema to access the data. To ensure that the service remains available to all users, EPA also makes effort to identify and block unauthorised attempts to upload or change information on their website.

### ToxCast

There is a huge data gap between environmental chemicals and their associated toxicity information. This is because of the expense and length of time required to conduct animal testing to obtain such toxicity data. Traditional animal testing provides very limited information on mechanism of action (MOA). MOA is a sequence of events from the absorption of a compound into a living organism to toxic outcome and it is crucial to predicting toxicity in humans. Therefore, there is a pressing need to screen the large backlog of chemicals for their potential toxicity and, ultimately, their contribution to human diseases [37]. Inspired by this, EPA NCCT launched the ToxCast Project [38,39] in 2007, in an attempt to develop an effective approach for prioritising the toxicity testing of a large number of environmental chemicals at low cost. The major goals of ToxCast are [37]:

• to detect *in vitro *assays which can reliably indicate alterations in biological processes that may lead to adverse health effects,

• to improve *in vivo *toxicity prediction by developing signatures or computational models from multiple *in vitro *bioassays, together with calculated and available chemical properties, rather than just using a single assay or chemical structure alone,

• to speed up the screening of the large number of untested environmental chemicals by the use of detected signatures from *in silico *and *in vitro *data.

As stated in the data governance framework (as shown in Figure 1), related domains are discussed as follows:

• **data accuracy: **it is claimed by EPA that the data included in the ToxCast project has been subjected to internal technical review and approved for research use. In most cases, the data has also undergone the external peer-review and publication in scientific journals [39]. In addition, the collection of ToxCast Phase I chemicals is carefully chosen subject to the availability of high quality, guideline-based animal toxicity data, and chemical property space coverage. The selected compounds represent the chemical space well and there are only a few compounds with extreme property values. In terms of updates, subsequent chemical analysis and verification of activity is still going on and the findings are added to the current data sources periodically.

• **data completeness: **in its initial stage, Phase I, ToxCast has profiled 320 chemicals, most of which are pesticides due to the availability of their extensive animal testing results, in over 400 high-throughput screening (HTS) endpoints using 9 different assay technologies. The current data source covers various chemical classes and diverse mechanisms of action and it has been deposited in PubChem. A total of 624 *in vitro *assay endpoints ranging from gene to entire organism have been measured for each chemical in ToxCast Phase I. The output of each chemical-assay combination was reported in terms of either half-maximal activity concentration (AC50) or lowest effective concentration (LEC) at which there was a statistically significant change from the concurrent negative control.

ToxCast Phase I is a proof of concept project to demonstrate its impact in various dimensions (e.g. chemical space, HTS assay data ("fast biology") and *in vivo *bioassay data ("slow biology")). Later phases will expand its impact to broader coverage of chemical space (over thousands of chemicals) and employ classes to validate the predictive toxicity signatures which built in Phase I. Phase II is currently screening 1,000 chemicals from a wider range of sources, including industrial and consumer products, food additives and drugs [39].

• **data integrity: **it is important to note that ToxCast includes *in vivo *data for ToxCast chemicals and such information is stored in a relational database called ToxRefDB [40] which contains nearly $2 billion worth of animal toxicity studies (55,950 in total). With the growth of the ToxCast database, the confidence in building statistical and computational models to forecast potential chemical toxicity will increase. This will result in refinement and reduction of animal use for hazard identification and risk assessment.

In addition to this, ToxCast data is well integrated to many other EPA databases. The ToxCast toxicity testing results are also available in the EPA ACToR data warehouse. They can be easily searched and combined with other related testing results derived from other projects. Also, the Phase I chemical structures and associated toxicity data are integrated in SDF files and they are available for download at the DSSTox website.

• **metadata and its management: **due to the availability of ToxCast Phase I data in DSSTox, the associated metadata (see data governance discussion in DSSTox) for these included information is provided. The software packages used to calculate physical and chemical properties from a chemical structure are indicated in the ToxCast data sources. For those *in vitro *assays included in ToxCast Phase I, the associated information, including contractor/collaborator, assay type, date stamp, assays/endpoints and references (publications and websites), are also provided in the EPA ToxCast website. In addition, ToxRefDB is the relational database storing *in vivo *toxicity testing data of ToxCast Phase I chemicals. The study details including time-stamps and owners are recorded in ToxRefDB.

• **data availability: **the ToxCast data sets, including descriptions, supplemental data and associated publications are available from the U.S. EPA ToxCast website. The users are allowed to download them individually in zip files. More complete and detailed information of the ToxCast chemicals, including chemical structures and identifiers, can be found in the EPA DSSTox website. The DSSTox ToXCST files http://www.epa.gov/ncct/dsstox/sdf_toxcst.html can be downloaded in diverse formats, such as sdf, spreadsheet and pdf. In addition, the ToxRefDB makes it possible to link toxicity information with the HTS and genomic data of ToxCast within the ACToR system. Recently, a new interface to access all ToxCast data has been provided via ToxCastDB [41]. It delivers a clear and easy browse format, such that users can search and download ToxCast chemicals, assays, genes, pathways and endpoints more effectively.

• **data authorisation: **similar to other EPA databases, ToxCast provides full and open access to included data and no user registration is required. For details, readers can refer to DSSTox data authorisation discussion.

### ACToR

Tens of thousands of chemicals are currently in commercial use, but only a small portion of them have been adequately assessed for their potential toxicological risk due to the expensive and time consuming conventional chemical testing methods. As aforementioned, the ToxCast project has been developed by EPA to speed up the process in filling such data gaps. ToxCast is also a major driver of the development of the Aggregated Computational Toxicology Resource (ACToR) project [42]. It is difficult to find related information about a given chemical. ACToR is therefore developed to support the ToxCast screening and prioritisation effort.

An important goal of the ACToR project is to develop a publicly accessible and widely usable database that gathers toxicity data associated with a large number of environmental chemicals from multiple sources [18,42]. The key uses of ACToR are to support predictive toxicology in terms of computational analysis [42,43]:

• collect and combine data from different sources to construct training and validation data sources to support high-throughput chemical screening and prioritisation efforts.

• serve as a unique resource which links chemical structure with *in vitro *and *in vivo *assays to support the development of computational models.

• provide EPA and other regulatory agency reviewers with the decision making support for novel chemicals approval.

• support the workflow construction feature enabling users to build customised prioritisation of data capture, quality control and chemical prioritisation scoring tasks.

The related domains in the data governance framework (see Figure 1) are examined as follows:

• **data accuracy: **ACToR database itself does not provide any data curation, the accuracy of the included data totally depends on the original sources of the data. Therefore, to ensure high data accuracy, the sources of included data collections are carefully selected by ACToR and the sources' institutional information are well recorded.

• **data completeness: **currently, ACToR is made up of over 500,000 environmental chemicals and their associated toxicity data which were derived from more than 500 public data sources including various databases such us: DSSTox, ToxCast and ToxRefDB; NIH, PubChem and TOXNET. ACToR is focused mainly on capturing chemical structures, physical and chemical properties, *in vitro *bioassays and *in vivo *toxicity data. In terms of chemical structures, EPA DSSTox and PubChem are the two main sources for ACToR due to their high data quality and wide data coverage, respectively. Although ACToR currently does not include all toxicity data, it is designed to be flexible enough to incorporate new data from sources with different formats into the system in a straightforward manner.

• **data integrity: **ACToR itself is an integration of available chemical toxicity data from a large number of sources. It is common that chemical toxicity data are stored in incompatible formats and in many different locations. In the past, in order to retrieve all relevant information for a given chemical, users needed to come across diverse data sources and aggregate the results manually for use. With the rapid increase of available chemicals and data sources, such a task becomes impractical and even impossible for comprehensive data sources. ACToR aggregates large number of chemical toxicity data and makes the included data searchable by chemical information (e.g. chemical name, chemical structure and identifiers). The data collections are formatted and organised in a consistent manner within ACToR. The clear and flexible ACToR database schema facilitates users to search and query data from other chemical toxicity data sources including ToxRefDB, ToxCastDB, DSSTox, ExpoCastDB and others to be added in the future.

• **metadata and its management: **the institutional sources of data collections and assay information are well included in ACToR. The recorded metadata of data collections includes: details of the data collection, source ID, name, description, source type, number of substance, number of generic chemicals, number of assay results, and link-out which provides a direct connection to the external website of a given data collection. In addition, the study and assay time-stamps, source ID, name, units, value type, component type as well as owners are included in the original data source, such as ToxRefDB.

• **data availability: **one of the significant advantages of using ACToR is its availability. The included data is provided in an easy accessible and computable manner via its web interface. The ACToR system is implemented using 100% open-source software, MySQL for the database development, Perl for loading data and Java for web interface development. This enables the user to download the entire ACToR database for local analysis. In addition, the ACToR database covers a wide variety of data sources. In particular, due to the use of similar data schemas, all data in PubChem can be easily loaded into ACToR. This feature makes the ACToR database easily expandable and scalable in the future.

• **data authorisation: **similar to other EPA databases, ACToR provides full and open access to the included data and no user registration is required. For details, readers can refer to DSSTox data authorisation discussion.

### OpenTox

OpenTox is a modern predictive toxicology framework under development [10]. Different from other toxicity data sources, OpenTox provides easy access to not only the good quality data, but also a collection of various predictive toxicity applications. OpenTox is designed to support effective data exchange and accurate cross-organisational communication and collaboration. Its flexible architecture and modular design contribute to the development of customised predictive toxicology applications with respect to user requirements. Currently, OpenTox provides two applications for model development and toxicity estimation. *ToxPredict *allows users to predict a toxicity endpoint for a given chemical compound.

*ToxCreate *supports predictive model generation. Developed models within the OpenTox can be validated, reported according to the OECD principles [44] and published within the OpenTox framework. Detailed model reporting supports reliable judgements about model validation and gives the possibility to reproduce predictions.

Data access and management, model development, feature construction and selection are core components of the OpenTox framework. Thus, OpenTox supports the creation of dictionaries and ontologies, that describe the relations between chemical and toxicological data and experiments and to develop novel techniques for the retrieval and quality assurance of toxicological information [9].

Recalling the data governance framework in Figure 1, the related domains are discussed as:

• **data accuracy: **OpenTox provides data quality assessment by assessing validation labels to included data (e.g. 2D chemical structures). It allows for three types of data quality validation: automated, manual and global. For a given chemical compound, its chemical structures can be imported from different sources. Then, these structures are automatically compared and classified into the following groups and each group is associated with a predefined validation label:

- consensus ("OK") - all structures are identical,

- majority ("ProbablyOK" and "ProbablyError") - majority of identical structures,

- ambiguous ("Unknown") - there is no majority of equal structures,

- unconfirmed ("Unknown") - single source and no comparison available.

The assigned quality labels will be further reviewed by experts according to their knowledge and manual comparison with external sources. The global validation aggregates the validation labels which derived from automated and manual validations for a given data source. Opentox employs a numerical measure, "ProbablyError" rate, to indicate the overall quality of a given data source. Obviously, the lower "ProbablyError%" indicates the better quality of the data source.

• **data completeness: **OpenTox mainly focuses on publicly available toxicology data. As reported in [9], OpenTox framework currently includes data from ISS ISSCAN, IDEA AMBIT, JRC PRS, EPA DSSTox, ECETOC skin irritation, LLNA skin, and the Bioconcentration Factor (BCF). The additional informations for chemical structures has been collected from public sources such as Chemical Identifier Resolver, ChemIDplus, PubChem.

• **data integrity: **is a current challenge for OpenTox. An ontology and controlled vocabularies have been developed by OpenTox to integrate and organise multidisciplinary data (e.g. chemicals, experiments, and toxicity data). With the support of ontology, OpenTox is currently moving towards the development of the Resource Description Framework (RDF) representation to exchange data from various sources.

• **metadata and its management: **OpenTox database provides means to identify the original sources of the included data by indicating inventor name and reference. By doing this, the user is allowed to select the compounds of interest from a specified inventory. OpenTox also makes the latest updates of the data (e.g. updates of chemical structures and descriptor calculations) which become available. OpenTox includes enhanced metadata for algorithms, models and datasets that are managed by the ontology web service.

• **data availability: **toxicity data is currently publicly available and accessible via the OpenTox Representational State Transfer (REST) web services. The RESTful web service has been chosen because it allows for the combination of different services into multiple applications to satisfy diverse user requirements. Additionally, OpenTox offers workflow architecture that is understandable and easy interpretable to users.

• **data authorisation: **the OpenTox website has public sections that are read accessible by anyone with a web browser. Only the OpenTox Development area requires a user name/password registration and registration approval. Having recognised the importance of multi-level user access, OpenTox have considered different authentication and authorisation solutions for an initial implementation to grant access to protected resources. A set of REST operations (including authentication, authorisation, create policy, delete policy and etc) have been published in OpenTox API.

## Summary

While the above discussed public data sources are well developed, there nevertheless remain some gaps in the development of data governance framework to support predictive toxicology. Firstly, more and more data repositories have realised the importance of included data quality and their inherent impacts on QSAR modelling. In most of the reviewed data sources (except ActoR), data curation procedures are applied to ensure accuracy and completeness of included data. These features are important components in data quality assessment. However, the data quality checking is mostly based on human expertise. While there are techniques leading to the automated data quality assessment, the derived results still have to be validated by experts. Moreover, there is a lack of systematic and standard measures of data quality, including data completeness, accuracy and consistency. The current assessments are all based on internal measures and this causes difficulty in comparing data from different sources. A more interpretable and transparent assessment mechanism is highly desirable and such standard measures would also be used to visualise data quality and then to compare and rank different data resources.

Second, data provenance is another substantial issue in predictive toxicology. Provenance is important and valuable to understanding, aggregating and making use of data sources and scientific results. In the presented data governance framework, data provenance is a part of metadata and its management. As shown in this review, the information about data submission, curation and authors are included as metadata in different manners and formats. This makes tracking the provenance of data very challenging. The determination of data provenance procedure and representation requires coordination and cooperation between various data sources, and this is currently very difficult. Automated data quality assessment will provide a more systematic and analytical metadata representation and this could lead to simplified and unified data quality control processes.

Third, the publicly available data sources contain rich and valuable scientific data and they are also of great interest to commercial companies. Combining company in-house data with existing public data will help to discover more hidden ideas and knowledge. However, as shown in the review, although CEBS and OpenTox have recognised the importance and benefits of multi-level user access, this development is still at the early stage. Most public data sources currently do not allow multi-level user access. All (registered) users share the same authorisation schema to access the data. The lack of protection of private and sensitive data becomes a bottleneck which limits commercial companies to contribute their in-house data to public repositories. It is challenging but highly desirable to develop more flexible and customised data authorisation schemas which will allow multi-level user access to both in-house and public data resources.

## Conclusions

In this paper, the review of some of the widely used public data sources which support predictive toxicology has been presented with regard to a proposed data governance framework. Current predictive toxicology challenges such as data integration and data quality assessment were authors' motivations to look at the existing solutions. In this review, widely used and well-known data resources were chosen, but the choice was not exhaustive.

It has been well recognised that data quality inherently affects model development. With the increasing amount of varied toxicity data that comes from *in vivo -in vitro *studies, there is expected to be a boom in the number of predictive toxicity models. Datasets and models should not be considered in isolation. A more systematic and analytical metadata representation would help users to explore the relationships between datasets and models. This will lead to better management of information and knowledge captured from metadata and help users to choose the most appropriate model for a given task. For example, it would be interesting to investigate who has used which datasets previously and for what purpose, by analysing the captured information. As a result, the most predictive and popular models can be stored, managed and reused for future work. The most important aspect in this context is an extraction of relationships between a large number of objects (chemical compounds, datasets, models and users). This will lead to better management of information and knowledge captured from such predictive toxicology objects. Additionally, it will help the visualisation of the relationship among various objects and will support the utilisation of the existing information. Development of such a framework will support monitoring the model life cycle, automated model selection, chemical compound identification across various projects, and can lead to speeding up the process of chemical compound virtual screening.

To achieve this, the following actions are foreseen:

• Represent data and models as programmable objects and provide standards for their representations. Existing dataset and model representation schemas (e.g. ToxML, QSAR-ML and PMML) can be employed and extended.

• Enable users to leave comments. In addition, each object (including dataset, model and user) can be associated with a wiki web link to keep notes and historical changes.

• Introduce a score rating schema which will allow users to rate the overall quality and suitability of the datasets and models that they have used. The storage of such metadata (e.g. rating scores and comments) would help to reduce duplication of effort and provide suggestions for subsequent users.

• Introduce a model version control system, which will allow models to be continuously updated whilst providing robust provenance of model predictions.

The authors believe that applying data governance in building information warehouses will provide a good start for data and model quality control. The analytical measurement of object quality, object similarity and object relationships monitoring will make such a framework more trustworthy and transparent for users and regulatory bodies. Standards in data and model representation will allow for effective object categorisation and consistent supporting documentation. This will lead to easy access to high quality information. It will also reduce the cost of information management, and secure the use of available data. Designing and developing a novel data and model governance framework is an important piece of future work. The main idea is to provide a common formatting system for data generation, extraction, curation, model estimation and validation. This will involve extension and unification of existing solutions that are accepted by regulatory bodies, and introduction of new standards in predictive toxicology. It also opens a wide area for various interesting research questions such as data provenance tracking, data and model quality measurements and the capture of object relationships.

## Competing interests

The authors declare that they have no competing interests.

## Authors' contributions

XF proposed the described data governance framework and contributed to the data sources review and the drafting of the manuscript. AW participated in analysis for database review and helped to prepare the manuscript for this publication. DN and MR firstly proposed the concept of data and model governance. DN, MR and KT have been involved in the review discussions and participated in revising critically this manuscript and proof read the draft. All authors read and approved the final version of the manuscript.
